# Genetic variation in five genes important in telomere biology and risk for breast cancer

**DOI:** 10.1038/sj.bjc.6603934

**Published:** 2007-08-14

**Authors:** S A Savage, S J Chanock, J Lissowska, L A Brinton, D Richesson, B Peplonska, A Bardin-Mikolajczak, W Zatonski, N Szeszenia-Dąbrowska, M Garcia-Closas

**Affiliations:** 1 Division of Cancer Epidemiology and Genetics, Clinical Genetics Branch, National Cancer Institute, National Institutes of Health, Bethesda, MD 20892, USA; 2 Pediatric Oncology Branch, Center for Cancer Research, National Cancer Institute, National Institutes of Health, Bethesda, MD 20892, USA; 3 Division of Cancer Epidemiology and Genetics, Core Genotyping Facility, National Cancer Institute, National Institutes of Health, Gaithersburg, MD 20877, USA; 4 Department of Cancer Epidemiology and Prevention, Cancer Center and M Sklodowska-Curie Institute of Oncology, Warsaw, Poland; 5 Division of Cancer Epidemiology and Genetics, Hormonal and Reproductive Epidemiology Branch, National Cancer Institute, National Institutes of Health, Bethesda, MD 20892, USA; 6 Department of Epidemiology, Nofer Institute of Occupational Medicine, Łódź, Poland

**Keywords:** breast cancer, telomere, *TERT*, *TERF2*, haplotype, single nucleotide polymorphism

## Abstract

Telomeres, consisting of TTAGGG nucleotide repeats and a protein complex at chromosome ends, are critical for maintaining chromosomal stability. Genomic instability, following telomere crisis, may contribute to breast cancer pathogenesis. Many genes critical in telomere biology have limited nucleotide diversity, thus, single nucleotide polymorphisms (SNPs) in this pathway could contribute to breast cancer risk. In a population-based study of 1995 breast cancer cases and 2296 controls from Poland, 24 SNPs representing common variation in *POT1*, *TEP1*, *TERF1*, *TERF2* and *TERT* were genotyped. We did not identify any significant associations between individual SNPs or haplotypes and breast cancer risk; however, data suggested that three correlated SNPs in *TERT* (−1381C>T, −244C>T, and Ex2-659G>A) may be associated with reduced risk of breast cancer among individuals with a family history of breast cancer (odds ratios 0.73, 0.66, and 0.57, 95% confidence intervals 0.53–1.00, 0.46–0.95 and 0.39–0.84, respectively). In conclusion, our data do not support substantial overall associations between SNPs in telomere pathway genes and breast cancer risk. Intriguing associations with variants in *TERT* among women with a family history of breast cancer warrant follow-up in independent studies.

Telomeres, located at the ends of chromosomes, consist of long TTAGGG nucleotide repeats and an associated protein complex. Chromosome ends are protected from end-to-end fusion and degradation by this telomere complex, termed shelterin (de Lange, 2005). The TTAGGG repeats shorten with each cell division, and eventually reach a critical state, at which time cellular senescence and/or apoptosis is normally triggered (Rodier *et al*, 2005). Tumour cells may survive cellular crisis in the absence of chromosomal stability through the activation or inactivation of alternative pathways. Breast cancer fits the paradigm of dysfunctional telomere-induced genomic instability, because the transition of breast duct hyperplasia to ductal carcinoma *in situ* likely results from a period of telomere crisis (DePinho, 2000; Chin *et al*, 2004). As breast cancer progresses further to invasive and metastatic stages, telomere dysfunction and genomic instability become more apparent (Nishizaki *et al*, 1997; Buerger *et al*, 1999; Chin *et al*, 2004). As cells progress through the latter stages of carcinogenesis, telomeres become relatively stable. In addition, low-telomere DNA content was found to be an independent predictor of decreased survival in comparisons of breast cancer specimens to normal tissues (Chin *et al*, 2004; Fordyce *et al*, 2006).

Most genes involved in telomere biology are highly conserved between species and have limited nucleotide diversity in humans (de Lange, 2004; Savage *et al*, 2005). We hypothesized that common genetic variation (minor allele frequency (MAF) greater than 5%) in the form of single nucleotide polymorphisms (SNPs) in these genes could affect cancer risk. This hypothesis was investigated in a population-based case–control study of breast cancer study in Poland, in which we genotyped 24 common SNPs that captured most of the common genetic variation in five genes important in telomere biology. The studied genes included telomerase (TERT (protein name), *TERT* (*HUGO* gene name), 5p15.33) (Collins and Mitchell, 2002), telomerase-associated protein (TP1, *TEP1*, 14q11.2) (Poderycki *et al*, 2005), telomeric repeat-binding factor 1 (TRF1, *TERF1*, 8q13) (Smogorzewska *et al*, 2000), telomeric repeat-binding factor 2 (TRF2, *TERF2*, 16q22.1) (Chong *et al*, 1995; Broccoli *et al*, 1997) and protection of telomeres 1 (POT1, *POT1*, 7q31.33) (Baumann and Cech, 2001).

## MATERIALS AND METHODS

### Study population

The design of this population-based breast cancer case–control study has been described (Garcia-Closas *et al*, 2006a). Eligible cases included women aged 20–74 years who were Polish residents of either Warsaw or Łódź with pathologically or cytologically confirmed *in situ* or invasive breast cancer, newly diagnosed in 2000–2003. An estimated 90% of eligible cases were identified through a rapid identification system at five participating hospitals. Information from Cancer Registries was used to identify the remaining 10% of eligible breast cancer cases. Eligible control subjects were residents of Warsaw and Łódź who did not have a history of breast cancer at enrollment. Controls were randomly selected from population lists, and frequency-matched to breast cancer cases by city and 5-year age groups. Women provided a personal interview on known and suspected risk factors. Venous blood samples were collected by a trained nurse. The study protocol was reviewed and approved by local and National Cancer Institute (NCI) Institutional Review Boards. All participants provided written informed consent. Of the 3037 eligible cases and 3639 eligible controls identified, 2386 (79%) cases and 2502 (69%) controls agreed to participate in the personal interview. The present study is limited to women with blood DNA samples: 1995 cases (6% *in situ*) and 2296 controls, which represented 84 and 94%, respectively, of the study population.

### Laboratory methods

Genomic DNA for genotype analyses was isolated from buffy coat or whole blood samples using the Autopure LS® DNA Purification System (Gentra Systems Inc., Minneapolis, MN, USA). Twenty-four SNPs in *POT1*, *TEP1*, *TERF1*, *TERF2*, and *TERT* were genotyped by investigators blinded to case–control status, using TaqMan or MGB Eclipse platforms at the Core Genotyping Facility of the Division of Cancer Epidemiology and Genetics, NCI (Table 1). Assay conditions are available at http://snp500cancer.nci.nih.gov (Packer *et al*, 2006). When possible, rs numbers based on the dbSNP database are indicated (http://www.ncbi.nlm.nih.gov/SNP). If an rs number has not yet been assigned, an E number (e.g. E3675_301) is provided, based on nomenclature from the SNP500Cancer project (Packer *et al*, 2006). Single nucleotide polymorphism locations were determined using the guidelines of the Human Genome Variation Society (den Dunnen and Antonarakis, 2001).

A total of 100 duplicate DNA pairs were ⩾98% concordant for each SNP with the exception of *TERF1* IVS9-163T>C (rs3863242, 97%) and *TERT* Ex2-659G>A (rs2736098, 94%). Genotypes were called for >98% of all SNPs. Genotype frequencies for all loci were in Hardy–Weinberg equilibrium among controls.

### Single nucleotide polymorphism selection

Initial SNP selection criteria included MAF greater than 5% in Caucasians from SNP500 Cancer (*n*=31), even spacing across the gene, SNPs with potential functional implications and/or patterns of nucleotide diversity and linkage disequilibrium (LD) previously determined through extensive re-sequence analysis (Savage *et al*, 2005; Packer *et al*, 2006) and assay availability at the time of SNP selection. The SNPs selected using these criteria were evaluated as haplotype-tagging SNPs compared with all common SNPs identified in the prior re-sequence analysis using tagSNPs (Stram, 2004) and TagZilla (http://tagzilla.nci.nih.gov/). *R*^2^_H_ was the pairwise correlation coefficient between SNPs determined by these programs. SNPs with *R*^2^_H_ ⩾0.8 were considered highly correlated.

*TEP1* (54 exons, 40.7 kilobase pairs (kbp)) has minimal LD and eight common SNPs in the 31 SNP500 Caucasians. The five *TEP1* SNPs genotyped (Table 1) gave an *R*^2^_H_ of 0.84, indicating representative coverage of common genetic variation across *TEP1*. *TERF1* (10 exons, 15.3 kbp) has very limited nucleotide diversity with only four common SNPs in SNP500 Caucasians between introns 7 and 9 (Savage *et al*, 2005). Three of these SNPs were genotyped and very good correlation for the fourth SNP was noted, *R*^2^_H_=1.0. *TERF2* (10 exons, 30.3 kbp) has only four common SNPs between introns 1 and 8 and a very small common haplotype block between introns 6 and 7 (Savage *et al*, 2005). *TERF2* IVS6+27G>A and IVS7-42T>C were highly correlated with the other SNP in this block, *TERF2* IVS8+95T>C (E3675_301) (*R*^2^_H_>0.8), but did not cover the SNP in intron 1 (*TERF1* IVS1-5C>T, E5055_301), which only had a MAF of 5% in SNP500 Caucasians. Studies of genetic variation in *TERT* (41.9 kbp, 16 exons) are complex due to low nucleotide diversity and limited LD (Savage *et al*, 2005). The 10 SNPs genotyped in our study spanned 43 kbp from −1654A>G to Ex16+203C>T and were representative of common genetic variation, *R*^2^_H_=0.63. We were unable to genotype *TERT* Ex14+7C>T (E3661_301, H1001H) due to lack of assay availability, which would have increased the *R*^2^_H_ to 0.83; however, we did genotype Ex16+203C>T (rs2853690), which was only 1776 bp 3′ of *TERT* Ex14+7C>T. The four SNPs genotyped in *POT1* (17 exons, 74.7 kbp) spanned 73.1 kbp (−1386G>A through IVS13-98T>G), a region with strong LD and 11 common SNPs in SNP500 Caucasians (Savage *et al*, 2005). These SNPs (Table 1) were good representatives of common genetic variation across *POT1*, *R*^2^_H_=1.0.

### Statistical analyses

Odds ratios (OR) and 95% confidence intervals (CI) from logistic regression models with dummy variables for matching factors (age in 5-year categories and study site (Warsaw or Łódź)) were used to estimate relative risks for the genotypes examined. The association between genotypes and breast cancer risk was tested using a 2 degrees of freedom (df) likelihood ratio test and a trend test. Heterogeneity of genotype ORs among groups of women defined by age categories and family history of breast cancer in first-degree relatives were evaluated by introducing interaction terms in logistic regression models. A positive family history was defined for women reporting one or more first-degree relatives diagnosed with breast cancer in the study questionnaire. An additive genetic model was assumed in interaction analyses. Age was considered as a continuous variable in tests for genotype–age interactions. Haplotypes were constructed for cases and controls using PHASE v2.1 (Stephens *et al*, 2001; Stephens and Donnelly, 2003) and HaploStats (Lake *et al*, 2003). The global case–control permutation test was performed using PHASE v2.1 (Stephens *et al*, 2001; Stephens and Donnelly, 2003). HaploStats (Lake *et al*, 2003) was used also to determine the global score *P*-value, haplotype frequencies, ORs and 95% CIs.

## RESULTS

Most cases (74%) and controls (69%) in the study were postmenopausal, and cases were diagnosed at an average age (standard deviation) of 56 (±10) years. The established risk factors were associated with breast cancer risk in comparable direction with similar estimates of magnitude reported by others (Garcia-Closas *et al*, 2006b).

Case–control analyses showed no statistically significant associations between the 24 SNPs in *TEP1*, *TERF1*, *TERF2*, *TERT* and *POT1* and risk of breast cancer (Table 1). Specific haplotypes derived from the evaluated SNPs were also not associated with increased risk of breast cancer in this study (data not shown). There were no statistically significant associations among age, SNP and breast cancer risk (Supplementary Table 1).

Case–control analyses suggested inverse associations between homozygous variants of *TERT* and breast cancer risk at two SNP sites, *TERT*-1654A>G (OR 0.85, 95% CI 0.72–1.02) and *TERT* Ex2-659G>A (A305A) (OR 0.76, 95% CI 0.58–1.00) (Table 1). The inverse association of *TERT* Ex2-659G>A (A305A) and two other linked *TERT* SNPs appeared to be limited to individuals with a family history of breast cancer in first-degree female relatives, −1381C>T (OR 0.73, 95% CI 0.53–1.00), −244C>T (OR 0.66, 95% CI 0.46–0.95), and Ex2-659G>A (A305A) (OR 0.57, 95% CI 0.39–0.84) (Table 2 and Supplementary Table 2). These SNPs were not significantly related to family history of cancer among the control population, and analyses of breast cancer cases with a family history of breast cancer compared with all controls, regardless of family history, produced similar results (data not shown). These three SNPs appeared to be in LD by D′, but only −244C>T and Ex2-659G>A were strongly correlated with *R*^2^_H_ of 0.79. *TERT*-1381C>T, −244C>T, and Ex2-659G>A had high pairwise D′ values, but the *R*^2^_H_ showed that only −244C>T and Ex2-659G>A were highly correlated. This suggests that the associations seen in *TERT* −1381C>T may not be related to the effects of LD between this SNP, −244C>T and Ex2-659G>A. However, the statistical association seen in −244C>T and Ex2-659G>A could be because they are highly correlated, and in effect, measure the same risk marker.

Haplotype analyses were performed for all SNPs studied in *TERT* and for each of the two major haplotype blocks in *TERT* (block 1: −1654A>G, −1381C>T, −967T>C, −244C>T and Ex2-659G>A, block 2: IVS10+269C>T and Ex16+203C>T). There were no significant associations for haplotypes in the primary case–control analysis (data not shown). However, a block 1 haplotype (ATCCA) in *TERT* was associated with protection from breast cancer when only individuals with a family history of breast cancer were studied (OR 0.61, 95% CI 0.38–0.97, *P*=0.034).

In addition, women with a family history also showed a borderline statistically significant positive association between *TERF2* IVS-42T>C variant alleles and breast cancer risk (OR 1.57, 96% CI 0.97–2.55, *P* interaction 0.06). No other associations were significantly modified by family history of breast cancer (Supplementary Table 2).

## DISCUSSION

To our knowledge, this is the first study to investigate genetic variation within genes important in telomere biology (*POT1*, *TEP1*, *TERF1*, *TERF2* and *TERT*) and breast cancer risk. The SNPs genotyped were representative of common genetic variation across the genomic region of interest, and showed no significant overall associations with breast cancer risk. However, data suggested association between variants in *TERT* among women with a positive family history of breast cancer.

*TERT* Ex2-659G>A showed a borderline statistically significant association with a reduced risk of breast cancer in analysis of all cases and controls, which appeared to be stronger for individuals with a family history of breast cancer. Similar associations of two other SNPs, −1381C>T and −244C>T, in individuals with a family history of breast cancer were also noted. *TERT* −244T>C was noted to have increased telomerase activity related to the T allele in a recent study of non-small cell lung cancer (Hsu *et al*, 2006). *TERT* −1381C>T also appears to be a functional SNP. Studies of promoter function at this site (noted at −1327 by the authors, but with the same rs number, rs2735940) suggested longer telomere length in with TT homozygotes compared with CC (Matsubara *et al*, 2006). Our findings suggested that variants in *TERT* could have an effect in individuals already at increased genetic risk of breast cancer, although the number of individuals with a family history of breast cancer was small.

*TERF2* IVS6+27G>A (E3673_301) was also associated with a reduced risk of breast cancer in individuals with a family history of breast cancer, however, the functional significance of the SNP is unknown. It does not appear to affect an intron–exon splice site (Conde *et al*, 2004).

The SNPs evaluated in this study were chosen based on previous knowledge of common genetic variation resulting from re-sequence analysis, captured most of the common variation in the five studied genes (i.e. *POT1*, *TEP1*, *TERF1*, *TERF2* and *TERT)*, and could be related to breast cancer risk based on the role suggested for telomere biology in this disease (Baykal *et al*, 2004; Wacholder *et al*, 2004; Savage *et al*, 2005). Although associations with less common SNPs are possible, our data indicate that common variation in these genes is unlikely to substantially affect overall breast cancer risk. The associations of *TERT* −1381C>T, −244C>T, Ex2-659G>A and the corresponding haplotype in individuals with a family history of breast cancer are intriguing and warrant follow-up in independent studies.

## Figures and Tables

**Table 1:** Association between 24 single nucleotide polymorphisms in five genes important in telomere biology and breast cancer risk among cases and controls

			**Controls**		**Cases**						
**Gene**	**SNP^a^**	**Genotype**	***N***	**%**	***N***	**%**	**OR**	**95% CI**		***P*-value**	***P* trend**
*TEP1*	Ex1-222 T>C	TT	1089	48	959	49	1.00				
	S116P	TC	972	43	831	42	0.97	0.86	1.11	0.68	
	Rs1760897	CC	203	9	183	9	1.02	0.82	1.27	0.84	0.93
	Ex4+51 C>A	CC	1514	67	1318	67	1.00				
	N307K	CA	657	29	572	29	1.00	0.87	1.14	0.96	
	rs1760898	AA	89	4	75	4	0.96	0.70	1.32	0.80	0.85
	IVS13+84T>C	TT	795	35	712	36	1.00				
	rs872072	TC	1078	47	928	47	0.97	0.84	1.10	0.61	
		CC	413	18	337	17	0.91	0.77	1.09	0.32	0.32
	Ex24+49 T>C	TT	625	28	503	26	1.00				
	S1195P	TC	1096	48	967	49	1.10	0.95	1.27	0.22	
	rs1760904	CC	540	24	495	25	1.14	0.97	1.35	0.12	0.12
	Ex45+36 G>A	GG	1433	63	1279	64	1.00				
	V2214I	GA	760	33	616	31	0.91	0.79	1.03	0.14	
	rs1713449	AA	88	4	92	5	1.18	0.87	1.60	0.28	0.66
											
*TERF1*	IVS7+82C>T	CC	1360	60	1146	58	1.00				
	E3663_301	CT	812	36	731	37	1.07	0.94	1.21	0.31	
		TT	106	5	106	5	1.19	0.89	1.57	0.24	0.15
	IVS8-124G>A	GG	983	44	836	43	1.00				
	rs2306494	GA	1017	45	885	45	1.02	0.90	1.17	0.72	
		AA	254	11	225	12	1.05	0.86	1.28	0.65	0.61
	IVS9-163T>C	TT	754	32	740	34	1.00				
	rs3863242	TC	1152	49	1060	48	0.93	0.82	1.06	0.30	
		CC	437	19	401	18	0.93	0.79	1.11	0.43	0.35
											
*TERF2*	IVS6+27G>A	GG	1603	70	1389	70	1.00				
	E3673_301	GA	612	27	535	27	1.01	0.88	1.16	0.88	
		AA	63	3	50	3	0.92	0.63	1.34	0.66	0.90
	IVS7-42T>C	TT	1081	47	894	45	1.00				
	rs251796	TC	960	42	873	44	1.10	0.97	1.25	0.13	
		CC	242	11	218	11	1.09	0.89	1.34	0.39	0.17
											
*TERT*	−1654A>G	AA	702	31	664	33	1.00				
	rs2736109	AG	1132	50	963	49	0.90	0.78	1.03	0.13	
		GG	443	19	357	18	0.85	0.72	1.02	0.08	0.06
	−1381C>T	CC	695	29	634	29	1.00				
	rs2735940	CT	1167	49	1121	51	1.05	0.92	1.20	0.46	
		TT	498	21	447	20	0.98	0.83	1.15	0.78	0.87
	−967T>C	TT	1671	73	1409	72	1.00				
	rs7712562	TC	556	24	510	26	1.09	0.94	1.25	0.24	
		CC	47	2	47	2	1.17	0.77	1.76	0.46	0.18
	−244C>T	CC	1224	54	1095	55	1.00				
	rs2853669	CT	900	39	766	39	0.95	0.84	1.08	0.42	
		TT	158	7	124	6	0.87	0.68	1.11	0.27	0.22
	Ex2-659G>A	GG	1313	58	1171	60	1.00				
	A305A	GA	811	36	699	36	0.97	0.85	1.10	0.59	
	rs2736098	AA	141	6	97	5	0.76	0.58	1.00	0.05	0.11
	IVS2-4601C>T	CC	1082	47	915	46	1.00				
	rs2736099	CT	957	42	857	43	1.05	0.93	1.20	0.42	
		TT	241	11	212	11	1.04	0.84	1.27	0.73	0.51
	IVS2-4455C>T	CC	890	39	738	37	1.00				
	rs2853677	CT	1062	47	950	48	1.08	0.94	1.23	0.27	
		TT	330	14	294	15	1.07	0.89	1.29	0.48	0.34
	IVS3-24T>C	TT	1731	76	1495	75	1.00				
	rs13167280	TC	518	23	460	23	1.03	0.89	1.19	0.71	
		CC	36	2	31	2	0.99	0.61	1.61	0.97	0.77
	IVS10+269C>T	CC	936	41	818	41	1.00				
	rs2075786	CT	1062	47	918	46	0.99	0.87	1.13	0.93	
		TT	283	12	244	12	0.99	0.82	1.21	0.95	0.93
	Ex16+203C>T	CC	1660	73	1454	74	1.00				
	rs2853690	CT	561	25	467	24	0.95	0.82	1.09	0.45	
		TT	49	2	43	2	1.00	0.66	1.52	0.99	0.55
											
*POT1*	−1386G>A	GG	966	42	851	43	1.00				
	E5047_301	GA	1055	46	913	46	0.98	0.86	1.11	0.74	
		AA	256	11	221	11	0.98	0.80	1.20	0.84	0.76
	IVS6-33G>A	GG	968	43	847	43	1.00				
	rs7784168	GA	1052	46	906	46	0.98	0.86	1.12	0.77	
		AA	249	11	220	11	1.01	0.82	1.24	0.94	0.94
	IVS12-111G>A	GG	1260	53	1154	52	1.00				
	rs10263573	GA	914	39	897	41	1.07	0.95	1.21	0.25	
		AA	185	8	155	7	0.91	0.73	1.15	0.44	0.86
	IVS13-98T>G	TT	909	39	861	39	1.00				
	rs10250202	TG	1111	47	1026	47	0.97	0.86	1.10	0.65	
		GG	332	14	314	14	1.00	0.84	1.20	0.98	0.88

Abbreviations: N=number of individuals with genotype data; OR=odds ratio; CI=confidence interval, UK=unknown.

Differences between total number of cases and controls and subjects shown in table are due to missing genotype information.

a The genomic location of the SNP is determined using guidelines from the Human Genetic Variation Society (den Dunnen and Antonarakis, 2001). If an rs number from the NCBI's dbSNP database is not available, the SNP is designated by an E number from the NCI's SNP500Cancer database (http://snp500cancer.nci.nih.gov).

**Table 2:** Association between selected single nucleotide polymorphisms in *TERF2* and *TERT* and breast cancer risk among cases and controls, stratified by family history of breast cancer in first-degree female relatives

			**Homozygous common**		**Heterozygous**		**Homozygous variant**		**Per minor allele relative risk**				
**Gene**	**SNP**	**Family history**	**Controls**	**Cases**	**Controls**	**Cases**	**Controls**	**Cases**	**OR**	**95% CI**		***P*-value**	***P* interaction**
*TERF2*	IVS6+27G>A	No	1496	1243	592	482	60	46	0.97	0.86	1.10	0.67	
	E3673_301	Yes	107	146	20	53	3	4	1.57	0.97	2.55	0.07	0.06
													
*TERT*	−1654A>G	No	662	598	1067	857	415	324	0.92	0.85	1.01	0.09	
	rs2736109	Yes	40	66	65	106	28	33	0.86	0.63	1.18	0.35	0.67
	−1381C>T	No	661	557	1093	1001	469	412	1.02	0.94	1.11	0.63	
	rs2735940	Yes	34	77	74	120	29	35	0.73	0.53	1.00	0.05	0.04
	−244C>T	No	1159	971	843	694	148	116	0.97	0.88	1.07	0.56	
	rs2853669	Yes	65	124	57	72	10	8	0.66	0.46	0.95	0.03	0.05
	Ex2-659G>A	No	1243	1037	761	634	130	93	0.96	0.86	1.07	0.44	
	A305A rs2736098	Yes	70	134	50	65	11	4	0.57	0.39	0.84	0.004	0.01
	IVS2-	No	1023	810	898	768	227	201	1.06	0.97	1.17	0.22	
	4601C>T rs2736099	Yes	59	105	59	89	14	11	0.75	0.53	1.06	0.10	0.06

Differences between total number of cases and controls and subjects shown in table are due to missing genotype information.
