# Supplementary material for: Genetic variation in five genes important in telomere biology and risk for breast cancer
Source: Br J Cancer. 2007 Aug 14;97(6):832–6. doi: 10.1038/sj.bjc.6603934 (PMC2360388; doi:10.1038/sj.bjc.6603934)
Supplement: Supplementary Tables 1 and 2 [file 6603934x1.doc]

**Supplemental Table 1:** Association between 24 SNPs in five genes important in telomere biology and breast cancer risk stratified by age. Per allele and P interaction values were determined using age as a continuous variable.

|  |  |  | Homozygous common | | Heterozygous | | Homozygous variant | | Per allele | | | | P |
| --- | --- | --- | --- | --- | --- | --- | --- | --- | --- | --- | --- | --- | --- |
| Gene | SNP | Age | Cases | Controls | Cases | Controls | Cases | Controls | OR | 95% CI |  | P value | interaction |
| *TEP1* | S116P | > 50 | 632 | 770 | 549 | 640 | 130 | 130 |  |  |  |  |  |
|  |  | ≤ 50 | 327 | 319 | 282 | 332 | 53 | 73 | 0.7 | 0.4 | 1.2 | 0.19 | 0.19 |
|  | N307K | > 50 | 876 | 1037 | 378 | 437 | 49 | 58 |  |  |  |  |  |
|  |  | ≤ 50 | 442 | 477 | 194 | 220 | 26 | 31 | 0.8 | 0.4 | 1.4 | 0.39 | 0.39 |
|  | IVS13+84T>C | > 50 | 470 | 550 | 624 | 720 | 220 | 283 |  |  |  |  |  |
|  |  | ≤ 50 | 242 | 245 | 304 | 358 | 117 | 130 | 0.9 | 0.5 | 1.5 | 0.67 | 0.81 |
|  | S1195P | > 50 | 339 | 413 | 630 | 749 | 333 | 372 |  |  |  |  |  |
|  |  | ≤ 50 | 164 | 212 | 337 | 347 | 162 | 168 | 1.4 | 0.8 | 2.2 | 0.20 | 0.30 |
|  | V2214I | > 50 | 843 | 969 | 415 | 519 | 62 | 60 |  |  |  |  |  |
|  |  | ≤ 50 | 436 | 464 | 201 | 241 | 30 | 28 | 1.1 | 0.6 | 2.1 | 0.68 | 0.62 |
| *TERF1* | IVS7+82C>T | > 50 | 751 | 925 | 495 | 547 | 71 | 75 |  |  |  |  |  |
|  |  | ≤ 50 | 395 | 435 | 236 | 265 | 35 | 31 | 0.9 | 0.5 | 1.7 | 0.86 | 0.67 |
|  | IVS8-124G>A | > 50 | 555 | 663 | 588 | 694 | 151 | 172 |  |  |  |  |  |
|  |  | ≤ 50 | 281 | 320 | 297 | 323 | 74 | 82 | 1.0 | 0.6 | 1.7 | 0.91 | 0.97 |
|  | IVS9-163T>C | > 50 | 500 | 509 | 700 | 796 | 264 | 285 |  |  |  |  |  |
|  |  | ≤ 50 | 240 | 245 | 360 | 356 | 137 | 152 | 0.9 | 0.6 | 1.5 | 0.73 | 0.85 |
| *TERF2* | IVS6+27G>A | > 50 | 921 | 1094 | 357 | 412 | 35 | 40 |  |  |  |  |  |
|  |  | ≤ 50 | 468 | 509 | 178 | 200 | 15 | 23 | 0.7 | 0.3 | 1.3 | 0.23 | 0.23 |
|  | IVS7-42T>C | > 50 | 602 | 726 | 565 | 658 | 151 | 167 |  |  |  |  |  |
|  |  | ≤ 50 | 292 | 355 | 308 | 302 | 67 | 75 | 1.2 | 0.7 | 2.0 | 0.53 | 0.68 |

| *TERT* | -1654A>G | > 50 | 448 | 472 | 639 | 774 | 231 | 299 |  |  |  |  |  |
| --- | --- | --- | --- | --- | --- | --- | --- | --- | --- | --- | --- | --- | --- |
|  |  | ≤ 50 | 216 | 230 | 324 | 358 | 126 | 144 | 0.8 | 0.5 | 1.4 | 0.44 | 0.66 |
|  | -1381C>T | > 50 | 434 | 478 | 738 | 795 | 294 | 328 |  |  |  |  |  |
|  |  | ≤ 50 | 200 | 217 | 383 | 372 | 153 | 170 | 0.8 | 0.5 | 1.2 | 0.26 | 0.26 |
|  | -967T>C | > 50 | 947 | 1138 | 328 | 369 | 29 | 34 |  |  |  |  |  |
|  |  | ≤ 50 | 462 | 533 | 182 | 187 | 18 | 13 | 1.2 | 0.6 | 2.5 | 0.54 | 0.71 |
|  | -244C>T | > 50 | 742 | 831 | 490 | 617 | 85 | 102 |  |  |  |  |  |
|  |  | ≤ 50 | 353 | 393 | 276 | 283 | 39 | 56 | 0.8 | 0.4 | 1.3 | 0.32 | 0.42 |
|  | A305A | > 50 | 792 | 885 | 447 | 560 | 72 | 95 |  |  |  |  |  |
|  |  | ≤ 50 | 379 | 428 | 252 | 251 | 25 | 46 | 0.9 | 0.5 | 1.6 | 0.64 | 0.84 |
|  | IVS2-4601C>T | > 50 | 613 | 720 | 565 | 668 | 140 | 159 |  |  |  |  |  |
|  |  | ≤ 50 | 302 | 362 | 292 | 289 | 72 | 82 | 1.0 | 0.6 | 1.7 | 0.97 | 0.93 |
|  | IVS2-4455C>T | > 50 | 507 | 606 | 611 | 729 | 198 | 213 |  |  |  |  |  |
|  |  | ≤ 50 | 231 | 284 | 339 | 333 | 96 | 117 | 0.8 | 0.5 | 1.4 | 0.48 | 0.37 |
|  | IVS3-24T>C | > 50 | 988 | 1170 | 310 | 357 | 21 | 24 |  |  |  |  |  |
|  |  | ≤ 50 | 507 | 561 | 150 | 161 | 10 | 12 | 1.1 | 0.5 | 2.3 | 0.83 | 0.87 |
|  | IVS10+269C>T | > 50 | 540 | 640 | 610 | 711 | 162 | 199 |  |  |  |  |  |
|  |  | ≤ 50 | 278 | 296 | 308 | 351 | 82 | 84 | 0.8 | 0.5 | 1.3 | 0.34 | 0.34 |
|  | Ex16+203C>T | > 50 | 954 | 1128 | 325 | 376 | 30 | 40 |  |  |  |  |  |
|  |  | ≤ 50 | 500 | 532 | 142 | 185 | 13 | 9 | 0.9 | 0.4 | 1.8 | 0.72 | 0.80 |
| *POT1* | -1386G>A | > 50 | 565 | 657 | 610 | 725 | 144 | 163 |  |  |  |  |  |
|  |  | ≤ 50 | 286 | 309 | 303 | 330 | 77 | 93 | 0.9 | 0.5 | 1.5 | 0.75 | 0.78 |
|  | IVS6-33G>A | > 50 | 565 | 662 | 606 | 724 | 143 | 158 |  |  |  |  |  |
|  |  | ≤ 50 | 282 | 306 | 300 | 328 | 77 | 91 | 0.9 | 0.6 | 1.6 | 0.78 | 0.79 |
|  | IVS12-111G>A | > 50 | 780 | 850 | 575 | 613 | 111 | 139 |  |  |  |  |  |
|  |  | ≤ 50 | 374 | 410 | 322 | 301 | 44 | 46 | 1.2 | 0.7 | 2.1 | 0.48 | 0.49 |
|  | IVS13-98T>G | > 50 | 568 | 614 | 681 | 768 | 213 | 217 |  |  |  |  |  |
|  |  | ≤ 50 | 293 | 295 | 345 | 343 | 101 | 115 | 0.9 | 0.5 | 1.4 | 0.61 | 0.62 |

**Supplemental Table 2:** Association between 24 SNPs in five genes important in telomere biology and breast cancer risk stratified by family history of breast cancer among first degree female relatives.

|  |  |  | Homozygous common | | Heterozygous | | Homozygous variant | | Per allele | | | | P |
| --- | --- | --- | --- | --- | --- | --- | --- | --- | --- | --- | --- | --- | --- |
| Gene | SNP | Family history | Cases | Controls | Cases | Controls | Cases | Controls | OR | 95% CI |  | P value | interaction |
| *TEP1* | S116P | No | 862 | 1023 | 743 | 914 | 166 | 195 | 0.99 | 0.90 | 1.09 | 0.83 |  |
|  |  | Yes | 97 | 66 | 88 | 58 | 17 | 8 | 1.12 | 0.79 | 1.60 | 0.52 | 0.50 |
|  | N307K | No | 1189 | 1419 | 507 | 627 | 69 | 83 | 0.97 | 0.87 | 1.09 | 0.65 |  |
|  |  | Yes | 129 | 95 | 65 | 30 | 6 | 6 | 1.24 | 0.82 | 1.87 | 0.30 | 0.26 |
|  | IVS13+84T>C | No | 637 | 742 | 827 | 1022 | 311 | 390 | 0.96 | 0.88 | 1.05 | 0.40 |  |
|  |  | Yes | 75 | 53 | 101 | 56 | 26 | 23 | 0.97 | 0.71 | 1.34 | 0.86 | 0.95 |
|  | S1195P | No | 444 | 586 | 882 | 1035 | 435 | 509 | 1.07 | 0.97 | 1.16 | 0.16 |  |
|  |  | Yes | 59 | 39 | 85 | 61 | 60 | 31 | 1.13 | 0.84 | 1.52 | 0.41 | 0.70 |
|  | V2214I | No | 1145 | 1360 | 555 | 705 | 82 | 83 | 0.99 | 0.89 | 1.11 | 0.92 |  |
|  |  | Yes | 134 | 73 | 61 | 55 | 10 | 5 | 0.75 | 0.52 | 1.09 | 0.14 | 0.16 |
| *TERF1* | IVS7+82C>T | No | 1033 | 1278 | 649 | 771 | 97 | 97 | 1.07 | 0.96 | 1.19 | 0.21 |  |
|  |  | Yes | 113 | 82 | 82 | 41 | 9 | 9 | 1.15 | 0.79 | 1.66 | 0.47 | 0.73 |
|  | IVS8-124G>A | No | 748 | 925 | 803 | 954 | 195 | 244 | 1.01 | 0.92 | 1.11 | 0.81 |  |
|  |  | Yes | 88 | 58 | 82 | 63 | 30 | 10 | 1.18 | 0.85 | 1.64 | 0.32 | 0.37 |
|  | IVS9-163T>C | No | 656 | 712 | 957 | 1088 | 356 | 407 | 0.97 | 0.89 | 1.06 | 0.49 |  |
|  |  | Yes | 84 | 42 | 103 | 64 | 45 | 30 | 0.86 | 0.64 | 1.15 | 0.30 | 0.42 |
| *TERF2* | IVS6+27G>A | No | 1243 | 1496 | 482 | 592 | 46 | 60 | 0.97 | 0.86 | 1.10 | 0.67 |  |
|  |  | Yes | 146 | 107 | 53 | 20 | 4 | 3 | 1.57 | 0.97 | 2.55 | 0.07 | 0.06 |
|  | IVS7-42T>C | No | 813 | 1018 | 768 | 905 | 199 | 229 | 1.05 | 0.96 | 1.16 | 0.29 |  |
|  |  | Yes | 81 | 63 | 105 | 55 | 19 | 13 | 1.21 | 0.86 | 1.71 | 0.27 | 0.44 |

| *TERT* | -1654A>G | No | 598 | 662 | 857 | 1067 | 324 | 415 | 0.92 | 0.85 | 1.01 | 0.09 |  |
| --- | --- | --- | --- | --- | --- | --- | --- | --- | --- | --- | --- | --- | --- |
|  |  | Yes | 66 | 40 | 106 | 65 | 33 | 28 | 0.86 | 0.63 | 1.18 | 0.35 | 0.67 |
|  | -1381C>T | No | 557 | 661 | 1001 | 1093 | 412 | 469 | 1.02 | 0.94 | 1.11 | 0.63 |  |
|  |  | Yes | 77 | 34 | 120 | 74 | 35 | 29 | 0.73 | 0.53 | 1.00 | 0.05 | 0.04 |
|  | -967T>C | No | 1266 | 1574 | 458 | 524 | 44 | 44 | 1.09 | 0.96 | 1.24 | 0.17 |  |
|  |  | Yes | 143 | 97 | 52 | 32 | 3 | 3 | 1.01 | 0.65 | 1.59 | 0.95 | 0.76 |
|  | -244C>T | No | 971 | 1159 | 694 | 843 | 116 | 148 | 0.97 | 0.88 | 1.07 | 0.56 |  |
|  |  | Yes | 124 | 65 | 72 | 57 | 8 | 10 | 0.66 | 0.46 | 0.95 | 0.03 | 0.05 |
|  | A305A | No | 1037 | 1243 | 634 | 761 | 93 | 130 | 0.96 | 0.86 | 1.07 | 0.44 |  |
|  |  | Yes | 134 | 70 | 65 | 50 | 4 | 11 | 0.57 | 0.39 | 0.84 | 0.00 | 0.01 |
|  | IVS2-4601C>T | No | 810 | 1023 | 768 | 898 | 201 | 227 | 1.06 | 0.97 | 1.17 | 0.22 |  |
|  |  | Yes | 105 | 59 | 89 | 59 | 11 | 14 | 0.75 | 0.53 | 1.06 | 0.10 | 0.06 |
|  | IVS2-4455C>T | No | 656 | 844 | 849 | 994 | 273 | 311 | 1.07 | 0.97 | 1.17 | 0.16 |  |
|  |  | Yes | 82 | 46 | 101 | 68 | 21 | 19 | 0.81 | 0.58 | 1.13 | 0.21 | 0.11 |
|  | IVS3-24T>C | No | 1342 | 1629 | 410 | 488 | 29 | 35 | 1.01 | 0.89 | 1.16 | 0.84 |  |
|  |  | Yes | 153 | 102 | 50 | 30 | 2 | 1 | 1.13 | 0.70 | 1.84 | 0.61 | 0.66 |
|  | IVS10+269C>T | No | 746 | 882 | 826 | 1002 | 206 | 264 | 0.97 | 0.88 | 1.06 | 0.51 |  |
|  |  | Yes | 72 | 54 | 92 | 60 | 38 | 19 | 1.22 | 0.89 | 1.66 | 0.22 | 0.17 |
|  | Ex16+203C>T | No | 1313 | 1559 | 410 | 533 | 38 | 45 | 0.94 | 0.82 | 1.06 | 0.31 |  |
|  |  | Yes | 141 | 101 | 57 | 28 | 5 | 4 | 1.25 | 0.81 | 1.94 | 0.31 | 0.21 |
| *POT1* | -1386G>A | No | 760 | 905 | 822 | 995 | 198 | 244 | 0.98 | 0.89 | 1.08 | 0.71 |  |
|  |  | Yes | 91 | 61 | 91 | 60 | 23 | 12 | 1.08 | 0.77 | 1.51 | 0.65 | 0.59 |
|  | IVS6-33G>A | No | 757 | 908 | 815 | 993 | 197 | 239 | 0.99 | 0.90 | 1.09 | 0.84 |  |
|  |  | Yes | 90 | 60 | 91 | 59 | 23 | 10 | 1.14 | 0.81 | 1.60 | 0.45 | 0.43 |
|  | IVS12-111G>A | No | 1025 | 1191 | 805 | 855 | 144 | 177 | 1.03 | 0.93 | 1.13 | 0.60 |  |
|  |  | Yes | 129 | 69 | 92 | 59 | 11 | 8 | 0.85 | 0.60 | 1.21 | 0.37 | 0.32 |
|  | IVS13-98T>G | No | 774 | 856 | 925 | 1049 | 273 | 311 | 0.98 | 0.90 | 1.07 | 0.71 |  |
|  |  | Yes | 87 | 53 | 101 | 62 | 41 | 21 | 1.07 | 0.79 | 1.44 | 0.66 | 0.60 |
